# Identification of Geometric Features of the Corrugated Board Using Images and Genetic Algorithm

**DOI:** 10.3390/s23136242

**Published:** 2023-07-07

**Authors:** Maciej Rogalka, Jakub Krzysztof Grabski, Tomasz Garbowski

**Affiliations:** 1 Institute of Applied Mechanics, Poznan University of Technology, Jana Pawla II 24, 60-965 Poznan, Poland; maciej.rogalka@o2.pl; 2 Department of Biosystems Engineering, Poznan University of Life Sciences, Wojska Polskiego 50, 60-627 Poznan, Poland; tomasz.garbowski@up.poznan.pl

**Keywords:** corrugated board, flute, flute type, cross-section image, genetic algorithm

## Abstract

The corrugated board is a versatile and durable material that is widely used in the packaging industry. Its unique structure provides strength and cushioning, while its recyclability and bio-degradability make it an environmentally friendly option. The strength of the corrugated board depends on many factors, including the type of individual papers on flat and corrugated layers, the geometry of the flute, temperature, humidity, etc. This paper presents a new approach to the analysis of the geometric features of corrugated boards. The experimental set used in the work and the created software are characterized by high reliability and precision of measurement thanks to the use of an identification procedure based on image analysis and a genetic algorithm. In the applied procedure, the thickness of each layer, corrugated cardboard thickness, flute height and center line are calculated. In most cases, the proposed algorithm successfully approximated these parameters.

## 1. Introduction

Corrugated board is commonly used for shipping boxes, retail displays, and other packaging applications. It is lightweight, easy to handle, and can be printed with custom designs. Furthermore, corrugated board is recyclable and can be biodegradable, making it an eco-friendly choice for companies and consumers alike. It is a versatile and popular material used in the packaging industry [1,2]. It consists of a fluted sheet sandwiched between two flat linerboards, which provides strength and durability while also allowing for flexibility.

The fluted sheet in corrugated board is made by passing paper through a series of fluting rolls, which create the characteristic ridges and valleys that give corrugated board its name. The flutes come in different sizes, with larger flutes providing more strength and cushioning, and smaller flutes providing a smoother surface for printing.

The linerboards that make up the outer layers of corrugated board are typically made from kraft paper, which is a type of paper made from wood pulp. Kraft paper is known for its strength, durability and ability to resist tearing and puncturing, making it an ideal material for packaging.

There are several types of fluted sheets used in the production of corrugated board, each with its own characteristics and advantages. The most common types are the following:

- A-flute: A-flute is the largest and thickest type of flute, with a height of approximately 5 mm. It provides excellent cushioning and is commonly used for packaging heavier items, such as appliances and furniture.
- B-flute: B-flute has a height of approximately 3 mm and is the second most common type of flute. It is a versatile option that provides good cushioning and is often used for shipping boxes and retail displays.
- C-flute: C-flute has a height of approximately 4 mm and is the most common type of flute. It provides good cushioning and is a popular choice for shipping boxes and re-tail displays.
- E-flute: E-flute has a height of approximately 1.6 mm and is the thinnest type of flute. It provides a smooth surface for printing and is often used for retail displays and small boxes.
- F-flute: F-flute has a height of approximately 0.8 mm and is the newest type of flute. It provides excellent printing quality and is ideal for small boxes and retail displays.

**Figure 1:**
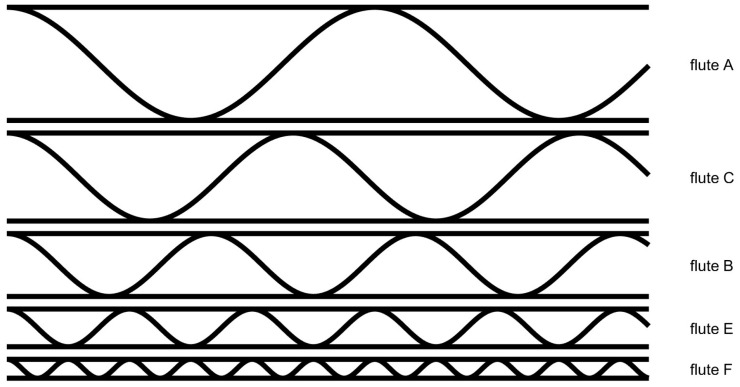
Flute types.

Each type of flute offers different benefits and is suitable for different types of pack-aging applications. Manufacturers can also combine different types of flutes to create custom corrugated boards that meet specific requirements for strength, cushioning and printing quality. Figure 1 shows, schematically, the differences between the flute types. One can notice that the main difference between them is their height.

The corrugated board can be warped during production and it can also be deformed after this process, or during its storage, transportation and use. The causes of these phenomena are related to changes in temperature and humidity or mechanical loads. One can distinguish two types of imperfections of the corrugated board, which are either global or local in nature. Beck and Ficherauer developed and described a model of systematic, large-scale deflections of the cardboard [3]. In this article, the authors paid more attention to local imperfections. The influence of imperfection size on the compressive strength of boxes made of the corrugated board was examined by Nordstrand in 1995 [4]. In 2004, the same author studied local imperfections by analyzing the nonlinear buckling of Rhodes and Harvey orthotropic plates [5]. The mechanical properties of corrugated cardboards, with imperfection during compression, were analyzed by Lu et al. [6]. An analytical study of double-walled corrugated cardboard bending was performed by Garbowski and Knitter-Piątkowska [7]. A method for the analysis of single-walled corrugated cardboard, introducing initial imperfections, was proposed by Mrówczyński et al. [8]. Cillie and Coetzee investigated corrugated cardboards with global and local imperfections under in-plane compression [9]. Recently, Mrówczyński and Garbowski presented a simple method for the calculation of the effective stiffness of corrugated board with geometrical imperfections using the finite element method and representative volumetric element [10].

Image processing is not so commonly used in corrugated board analysis. However, the most common example is related to creating a system for automatic waste sorting. For instance, Liu et al. developed a new model for garbage classification based on transfer learning and model fusion [11]. Similarly, Rahman et al. developed a classification for recyclable waste paper sorting using template matching [12]. Another application of the image processing algorithm is for the counting of the corrugated board layers. Cebeci applied some typical image processing operations for the automatic counting of the corrugated board [13]. Similarly, Suppitaksakul and Rattakorn applied a machine vision system and image processing techniques to count the corrugated board [14]. Later, Suppitaksakul and Suwannakit developed an algorithm for stitching corrugated board images [15].

In the literature, one can find analyses of cross-sectional geometry and classifications of various materials based on images. Caputo et al. applied the support vector machine to classify materials based on their images under various illumination and pose conditions [16]. Iqbal Hussain et al. applied the convolutional neural network, based on a pretrained network architecture ResNet-50, for the recognition and classification of woven fabrics [17]. Wyder and Lipson examined convolutional neural networks for the identification of the static and dynamic properties of cantilever beams based on their raw cross-section images [18]. Li et al. applied various deep learning techniques for analyzing the geometric features of a self-piercing riveting cross-section [19]. They showed that the SOLOv2 and Unet architectures give the best results. Ma et al. analyzed the geometrical features of crushed, thin-walled, carbon fiber-reinforced polymer tubes cross-sections [20].

Genetic algorithm is an optimization algorithm inspired by the process of natural selection and genetics [21]. These algorithms are used to solve complex problems by mimicking the principles of evolution, such as selection, crossover and mutation. The basic idea behind genetic algorithms is to create a population of individuals that represent potential solutions to a given problem. Each individual is encoded as a set of parameters, often called chromosomes or genomes, which can be considered as the genetic material. These chromosomes are subject to operations like selection, crossover and mutation, which emulate the genetic processes of reproduction and variation. The pioneer of genetic algorithms is John Henry Holland [22]. Genetic algorithms have been successfully applied to a wide range of problems in the areas of optimization, scheduling problems or artificial intelligence. They are particularly useful when dealing with complex, multi-dimensional search spaces, in which traditional optimization algorithms may have failed. Genetic algorithms have been also applied in some problems related to corrugated boards. Recently, Shoukat applied a genetic algorithm in combination with mixed integer linear programming for the problems of minimizing cost and greenhouse gas emissions during papermaking processes [23]. Hidetaka and Masakazu used a genetic algorithm to solve the scheduling problem of corrugated board production [24]. However, a genetic algorithm has not been applied yet to find the geometrical features of the corrugated board.

In this paper, the authors presented a new method for the analysis of corrugated board geometrical features. Equipment for the acquisition process of the corrugated board samples was designed and manufactured. Then, an algorithm for the analysis of the corrugated board geometrical features was proposed and implemented. The algorithm was based on image processing operations and the application of the genetic algorithm for finding the geometrical features of the flute. The presented methodology can be a first step for the automatic modelling of the corrugated board geometry based on its cross-section image.

## 2. Materials and Methods

In this section, the equipment designed and used for the acquisition of the corrugated board samples, as well as the algorithm proposed to identify geometrical parameters of the flute are described.

### 2.1. Device for the Acquisition of Corrugated Board Cross-Section Images

**Figure 2:**
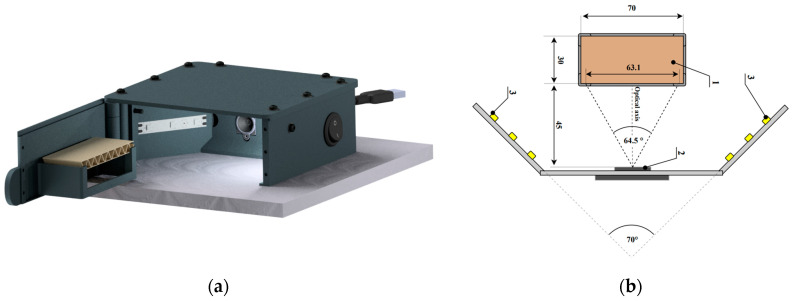
Device for corrugated board image acquisition: (**a**) visualization of the device; (**b**) layout diagram of the most important components of the device: 1—corrugated board sample; 2—camera; 3—LED strip.

In order to acquire the images of the corrugated board cross-section, a dedicated device was designed and manufactured. This device allowed us to record the images of the samples’ cross-sections under uniform acquisition conditions. Figure 2a shows a visualization of the device with an example of the corrugated board section. The sample holder is placed on the door, the opening of which allows for the free mounting of the sample. Closing the door and limiting its uncontrolled opening is ensured by neodymium magnets placed in the door frame. The camera is mounted on the device frame in such a way that its optical axis is directed perpendicularly to the plane of the sample face (Figure 2b). Two LED strips with a power of 4.8 W/m, mounted on the dividing wall, provide a uniform illumination of the photographed surface. Lighting is controlled manually by using a bistable key switch. Two wires are led out of the device, a power cable for the lighting module and a USB cable, which allows us to acquire the image from the camera and save it on the computer. The device was manufactured using 3D printing technology.

The system used the ArduCam B0197 camera with the autofocus function and the Sony IMX179 (1/3.2”) image sensor with a resolution of 8 MPx. The image from the camera was saved in JPEG format and had a maximum resolution of 3264 × 2448 pixels.

### 2.2. Algorithm for the Geometrical Feature Determination of the Corrugated Board

**Figure 3:**
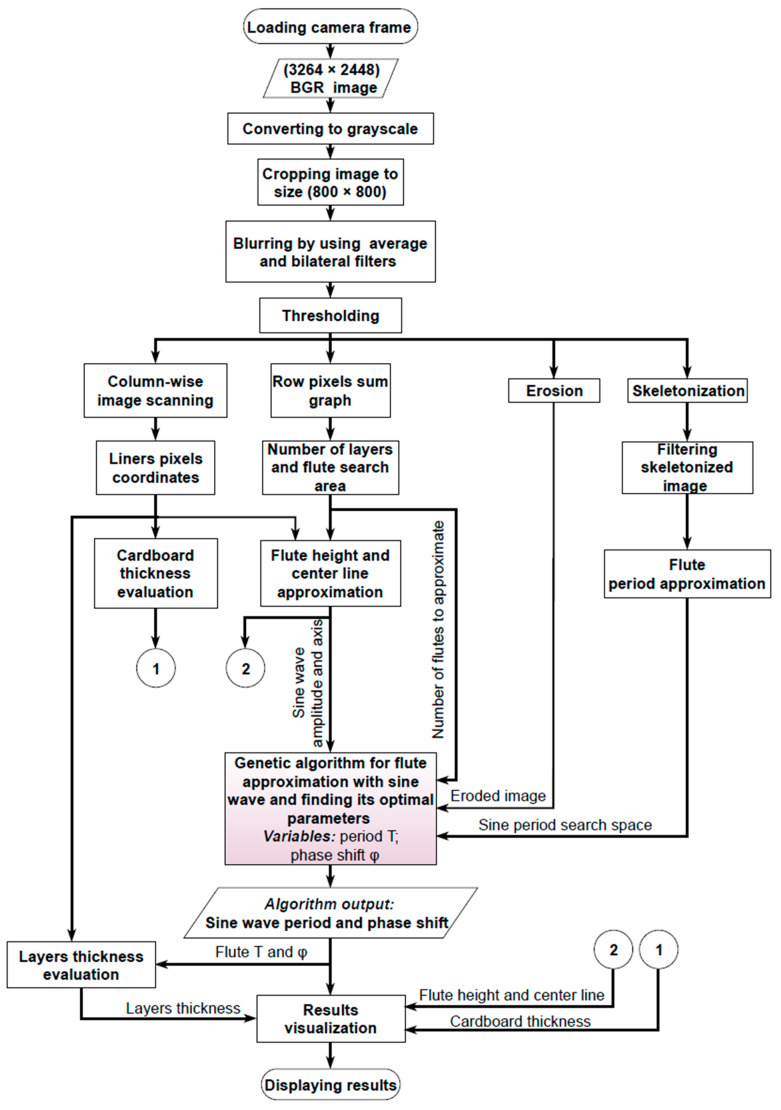
Flowchart of the proposed algorithm.

The flowchart of the proposed algorithm is presented in Figure 3. It consists of some preprocessing steps and parallel operations in which the geometrical features of the corrugated board, including its height, flute period and phase shift, liners and flute thickness, are determined. The algorithm has been implemented in Python language using the OpenCV library.

#### 2.2.1. Preprocessing Stage

**Figure 4:**
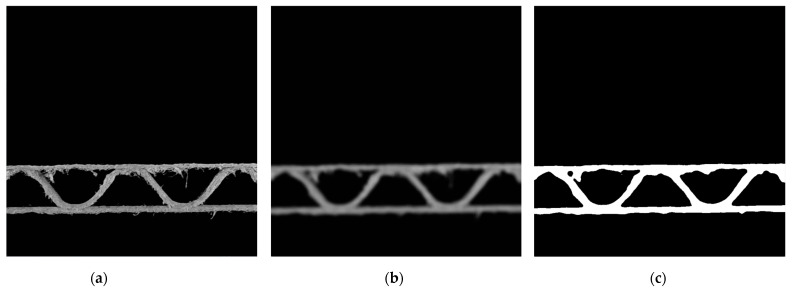
Results of the preprocessing stage: (**a**) a gray-scale cropped image of 800 × 800 pixels; (**b**) blurred image; (**c**) binary image.

At the preprocessing stage, a single frame from the camera was acquired. It was an RGB image with dimensions of 3264 × 2448 pixels. Then, it was converted to a grayscale image in the range <0, 255>. A fragment of the gray-scale image with dimensions of 800 × 800 pixels was cut out in the central acquisition area. An example of the results of these operations is shown in Figure 4a. In the next step, the image was blurred using two operations. At first, the averaging operation with a normalized box filter and with a kernel size of 3 × 3 was applied. Then, a bilateral filter was used. The result of these operations is presented in Figure 4b. It allowed us to remove small noise (small fibers) in the image related to the cutting of the sample. One can notice that the larger fibers are still visible in the image. The last step in the preprocessing stage was the lower threshold binarization. The threshold was equal to 75. All the parameters in the preprocessing stage were chosen empirically.

#### 2.2.2. Corrugated Cardboard Thickness Estimation

**Figure 5:**
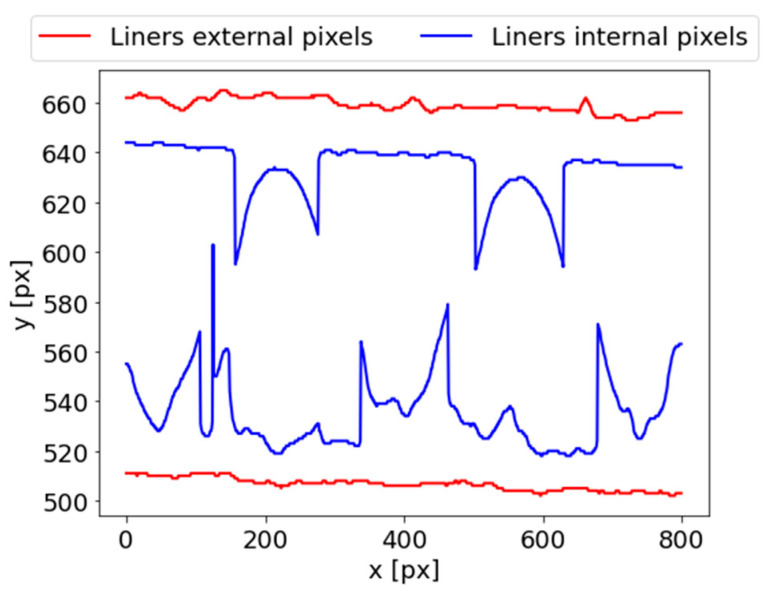
The results of the column-wise image scanning.

The purpose of the next stage was to estimate the corrugated cardboard thickness. This can be obtained by observing the boundary points of each corrugated cardboard layer in each column. This operation is called column-wise image scanning. Furthermore, it is necessary to estimate the thickness of the flute and liners in the next stages of the proposed algorithm. In the column-wise image scanning operation, each column of the binary image (Figure 4c) is analyzed. Starting from the top towards the bottom of the image, the first white pixel is searched. The y coordinate of this pixel is written in the ULEP matrix, which contains the external points of the upper liner. In a given column, after finding the first white pixel, the first black pixel is searched. It describes the background in the flute area. The y coordinate of this pixel is written in the ULIP matrix, which contains the internal points of the upper liner. A similar procedure can be applied for the determination of lower liner points. The only difference is in the direction of searching. For the lower liner, it should be performed starting from the bottom towards the top of the image. In this way, the matrices LLEP and LLIP are obtained, which denote the lower liner external points and lower liner internal points, respectively. The results of the findings of the internal and external points of the upper and lower liners are presented in Figure 5.

The corrugated cardboard height can now be obtained by calculating the average distance between the external points of the upper and lower liners. Thus, it can be expressed as

(1) $$d=\frac{1}{NC}\sum_{x=0}^{NC-1}\left|ULEP\left(x\right)-LLEP\left(x\right)\right|,$$

where x denotes the column index, and NC is the total number of columns. Thus, in this case (for the cropped image), NC=800.

After the column-wise image scanning operation, the external boundaries of the liners can be determined using the matrices ULEP and LLEP. Both boundaries can be approximated using linear functions based on the matrices of these coordinates:

(2) $$y_{U}=a_{U}x+b_{U},$$

(3) $$y_{L}=a_{L}x+b_{L},$$

where $a_{U}$ and $b_{U}$ denote the parameters of the upper liner approximation, while $a_{L}$ and $b_{L}$ are the parameters of the lower liner approximation.

#### 2.2.3. Corrugated Cardboard Center Line and Flute Height Estimations

**Figure 6:**
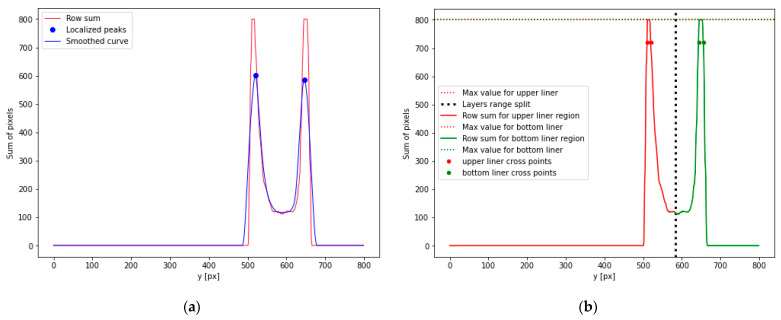
The row sum curve and localizations of the vertical positions of liners: (**a**) the row sum curve (red line) and the smoothed curve (blue line); (**b**) the upper (red) and lower (green) liners’ ranges.

In the next stage, the corrugated cardboard center line and flute height are estimated. The row sum curve of the binary image is determined in order to find the localization of liners. Now, the rows of the binary image are analyzed and the sum of white pixels at each row is calculated. The corrugated carboard sample is placed horizontally. Therefore, the maximums in the row sum curve are related to the liners’ localizations. Due to the flute and other disturbances, many local maximums can be observed in the row sum curve. Therefore, curve smoothing is applied using the Savitzky–Golay filter with 30 interpolation points and a polynomial of the first degree. In such a way, the number of local minima is limited. Furthermore, to find the additional conditions, the following were taken into account: the minimal distance between maximums, which was equal to 20, and the minimal value of the local maximum, which was equal to 0.4 of the global maximum value. The local minimums, found in such a way, are related to the vertical positions of liners (their y coordinates). This study was limited to 3-layered corrugated boards, which always have 2 liners and 1 flute. Thus, at this stage, two local maximums should be detected, as is depicted in Figure 6a. This figure shows the row sum curve and the result of its smoothing using the Savitzky–Golay filter. It is also worth noting that for both the ideal samples and the creased samples, the layers of the corrugated carboard can have different thicknesses and can be deformed in different ways, which affects the peak values, and it is possible that they can differ significantly in the values of the local maximums.

Now, the original non-smoothed row sum curve can be analyzed, see Figure 6b. It is divided into two ranges. The split of them is determined as a middle point between the two local maximums (black bold dashed line). In Figure 6b, the upper liner range is marked in red, while the lower liner range is marked in green. Within each of these ranges, the following steps are performed:

1. The local maximum is determined.
2. All the points in the range, which have values greater than 0.9 of the local maximum, and their boundary points are chosen. This is depicted in Figure 6b. The boundary points of these points, which have values greater than 0.9 of the local maximum value, are marked by bold dots within each range.
3. The distances between the boundary points are calculated and denoted as $b_{US}$ and $b_{BS}$ for the upper and bottom liners, respectively.

The boundary lines are used in the next steps for the limitation of the searching range. Based on the external boundaries of the liners expressed in Equations (2) and (3), the boundary lines limit the flute searching. They can be written in the following forms:

(4) $$y_{UBL}=a_{U}x+b_{U}+b_{US},$$

(5) $$y_{LBL}=a_{L}x+b_{L}-b_{LS}.$$

**Figure 7:**
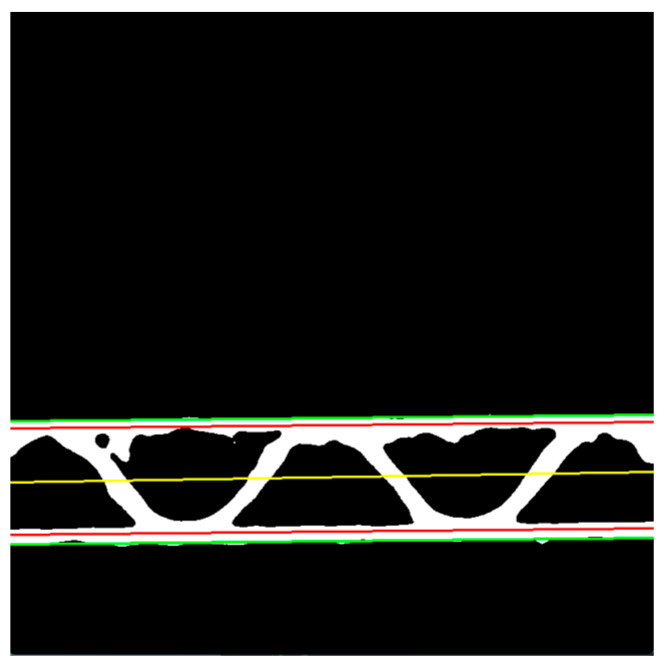
The binary image with external boundaries of the liners (green lines), boundary lines for limiting the flute searching (red lines), and the central line (yellow line).

These boundary lines are depicted in red in Figure 7, while the external boundary lines, expressed in Equations (2) and (3), are presented in green. The center line is approximated as a central line between these two red lines and can be expressed as

(6) $$y_{center}=a_{center}x+b_{center},$$

where $a_{center}$, $b_{center}$ are the parameters of the center line.

The height of the flute, which is equal to two times the amplitude of the sinusoidal function, can be approximated using the following formula:

(7) $$H=\frac{1}{NC}\sum_{x=0}^{NC-1}\left|y_{UBL}\left(x\right)-y_{LBL}\left(x\right)\right|=\frac{1}{NC}\sum_{x=0}^{NC-1}\left|\left(a_{U}-a_{L}\right)x+b_{U}+b_{US}-b_{L}+b_{LS}\right|.$$

#### 2.2.4. Limitations for the Flute Period Searching

**Figure 8:**
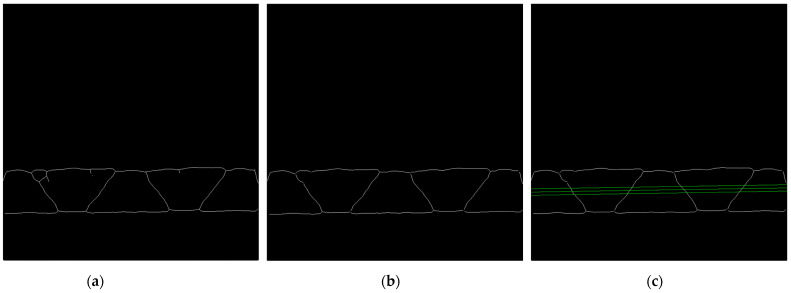
(**a**) Results of the skeletonization process; (**b**) results after skeletonization process and removing side branches; (**c**) 3 parallel green lines for period limitations.

In next step, the skeleton of the binary image is determined, see Figure 8a. There are some disturbances in the skeleton related to existence of the fibers coming from the cutting process of the samples. Due to the presence of these unfavorable side branches, which are formed due to the skeletonization of the binary image, the skeleton is additionally subjected to a filtering process. It consists of removing side branches when their contour length is less than 50 pixels, see Figure 8b.

The limitation for the range of period searching determined by the values $T_{min}$ and $T_{max}$ (the range of the period searching in pixels) is based on calculating the distances between the intersection points of the skeleton contours, with 3 parallel lines (parallel to the upper liner) drawn through the central flute area, see Figure 8c. Based on the averaged distances between successive intersection points and the maximum distance, the values $T_{min}=0.5{di}_{av}$, $T_{max}=3{di}_{max}$ are taken, where ${di}_{av}$ is the average distance between the intersection points for the 3 parallel lines and ${di}_{max}$ denotes the maximal value of the distances between these intersection points. When the skeleton has too many disturbances, making it impossible to determine these values, e.g., due to the presence of long branches or critical deformation of the sample, the values $T_{min}=50$, $T_{max}=800$ are adopted.

#### 2.2.5. Sinusoidal Function Parameters Searching Using Genetic Algorithm

In the searching process for the parameters of the flute, the following formula for its approximation was taken into account:

(8) $$y_{flute}=a_{center}x+b_{center}-A\sin\left(\varphi +\frac{2\pi }{T}x\right),$$

where the parameters $a_{center}$, $b_{center}$ and amplitude A=H/2 were determined in the previous stages of the proposed algorithm. The phase shift φ and the period T were determined using the genetic algorithm.

**Figure 9:**
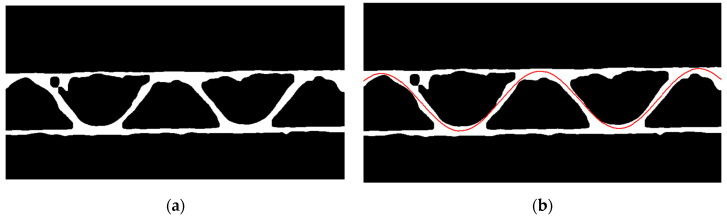
(**a**) The eroded image; (**b**) an example result of the optimization process using the genetic algorithm (red line).

As the input to the genetic algorithm, the eroded image of the binary image is treated, as presented in Figure 9a. Furthermore, the search for the phase shift φ and period T is limited by $\varphi_{min}=0$, $\varphi_{max}=2\pi$, $T_{min}$, and $T_{max}$. The objective function is the sum of the joint pixels for the eroded image and the function expressed in Equation (8) for the given φ and T.

The following parameters of the genetic algorithm were used:

- Maximal number of iterations: 500;
- Population size: 100;
- Mutation probability: 0.15;
- Elite group ratio: 0.01;
- Crossover probability: 0.2;
- Parents portion: 0.2;
- Crossover type: uniform.

An example of the genetic algorithm results is shown in Figure 9b.

#### 2.2.6. Estimation of the Corrugated Cardboard Layers Thicknesses

**Figure 10:**
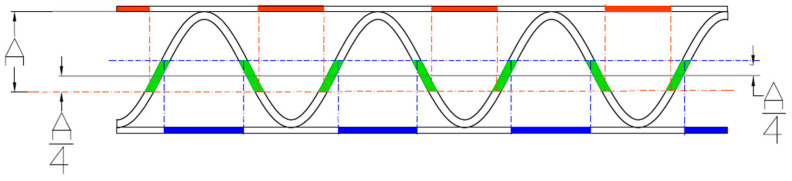
Schematic representation of the algorithm for determination of the liners and flute thicknesses – red, blue and green areas – regions for determination of the upper liner, lower liner and flute thicknesses, respectively.

The estimation of the thicknesses is schematically shown in Figure 10. The estimated position of the flute in the image allows us to choose the areas of the liners and flute in which the thickness of the papers can be measured. It allows us to avoid the ranges of the gluing between the liner and flute. In these regions, there are also lower numbers of disturbances related to the fibers coming from the sample cutting process. The area of the upper liner, where the thickness can be measured, is marked in red. A similar area for the bottom liner is marked in blue. The green color indicates the area in which the thickness of the fluting layer is determined.

**Figure 11:**
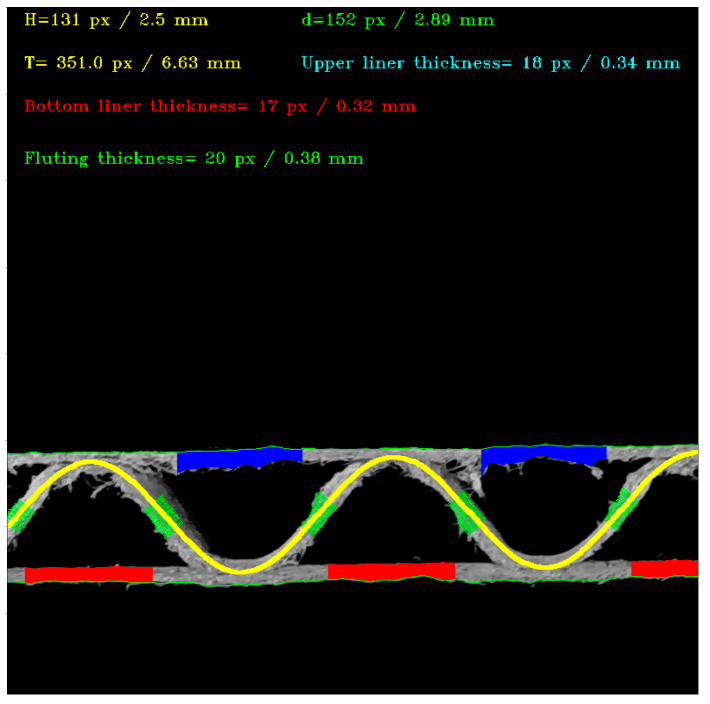
An example of the results obtained by applying the proposed algorithm.

These three areas were determined based on the sinusoidal function approximation determined in the previous step using the genetic algorithm. An example of the results is presented in Figure 11.

## 3. Results

The procedure presented above allowed us to identify the geometrical parameters of any single-wall corrugated board sample in a fully automatic manner. However, reliable results were obtained only for the samples with relatively small cross-section crushing and those cut in a way that does not damage the structure of individual layers.

**Figure 12:**
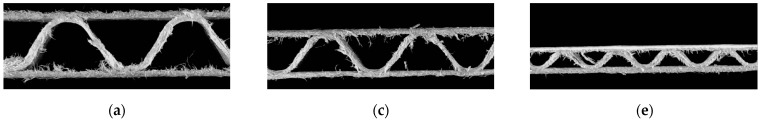

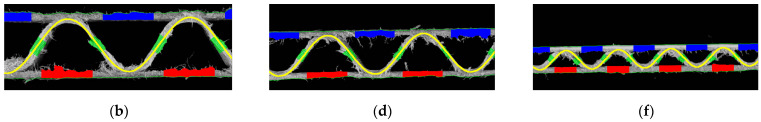
Visualization of the recognized fluting shapes of corrugated board: (**a**) flute C sample; (**b**) results obtained for the flute C sample; (**c**) flute B sample; (**d**) results obtained for the flute B sample; (**e**) flute E sample; (**f**) results obtained for the flute F sample.

Figure 12 shows the visualization of the recognized fluting shapes of corrugated board and the thickness of individual layers for three samples, i.e., for flute C, B and E. All the geometrical features of the single-ply corrugated cardboard cross-section identified by the procedure presented in this paper are summarized in Table 1. The results are presented both in the form of measured pixels and millimeters converted in the calibration process.

Each measurement method has a certain precision and limitations. In the case of the procedure presented here, the precision of the measurement, and thus the identification of the geometrical parameters of the corrugated board, is closely related to the quality of the provided sample. Any imperfections of the sample in the form of deformation of the corrugated layer as a result of crushing, shreds of fibers, or the inaccuracy of cutting directly affect the precision of the algorithms used for image analysis.

**Figure 13:**
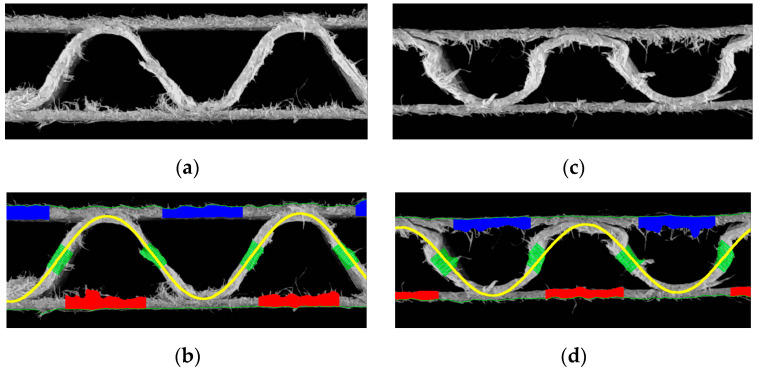
Examples of samples difficult to identify: (**a**) with many jagged edges; (**b**) results of identification of the sample with many jagged edges; (**c**) crushed sample; (**d**) results of identification of the crushed sample.

In Figure 13, it is easy to see that the precision of identifying both the thickness of individual papers and the shape of the corrugated layer depends on the quality of the sample. If the sample has many jagged edges, it is difficult to identify the thickness, while when the sample is crushed (on the right) and the shape of the fluting changes, the sinusoidal function approximation is still possible but does not reflect its real shape. In such cases, the identified period and wave height can only be used as auxiliary indicators.

Table 2 shows the identification results for two samples of the same cardboard (flute C), one of which (on the left) is not damaged in any way by creasing, while the other is damaged stochastically. Both samples are cut on a plotter with an oscillating knife, which means that the cut edges are not regular, and, in both cases, shreds of cellulose fibers represented an additional difficulty in the identification process.

**Figure 14:**
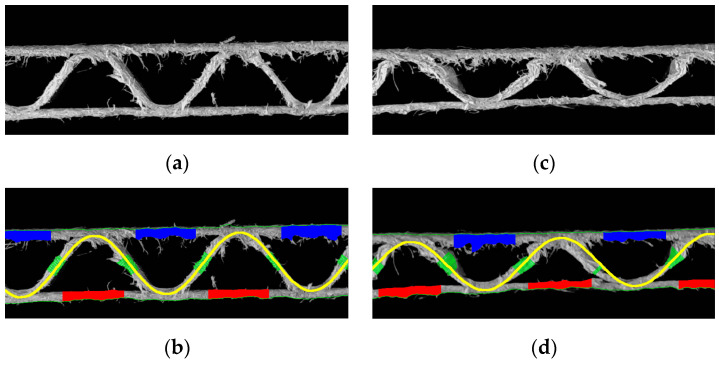
Flute B example: (**a**) cardboard without damage; (**b**) results of identification; (**c**) damaged sample; (**d**) results of identification.

Figure 14 shows two samples of corrugated board with flute B. Again, two cases are presented here–cardboard without damage (left) and with damage in the form of a crushed flute (right).

Table 3 contains all identified geometrical features of both corrugated board samples with the B-flute. It can be seen that in the case of cardboard with a lower flute, the identification of the shape of damaged cardboard is less error-prone, which makes the identified parameters of the corrugated layer more useful.

**Figure 15:**
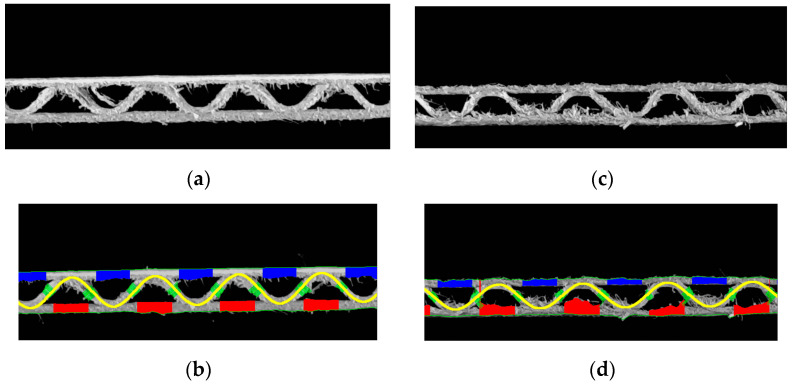
Flute E example: (**a**) cardboard without damage; (**b**) results of identification; (**c**) damaged sample; (**d**) results of identification.

The last example is shown in Figure 15, where two samples of corrugated board with E-flute are presented. This is the lowest flute of all the cases presented here; therefore, the crease in such cardboard is less visible (example on the right). As the geometrical dimensions of the cross-section are reduced, the measurement noise, which arises as a result of jagged edges, becomes more important and thus it is more difficult to obtain reliable values of the thickness of individual papers.

Table 4 presents the results of the identification of parameters for both samples of corrugated board with E-flute. Also, in this case, damage to the board in the form of creases is not as spectacular as in the case of cardboard with C-flute, which makes the identified parameters of the flute geometry more reliable.

The results presented in this section are only a small sample of all analyzed cases. The proposed identification procedure was tested on over 200 different cases. As already mentioned in the research campaign, both undamaged and damaged samples were used. All damages in the form of corrugated layer crushing were caused by completely stochastic processes with different forces and in different sample locations.

## 4. Discussion

The previous section presents representative examples of the operation of the algorithm for identifying the geometric parameters of cross-sections of various cardboard samples. Since, according to our knowledge, in the professional and scientific literature there are no similar attempts to identify the parameters describing the shape of the corrugated layer and the thickness of individual cross-section layers on real cardboard samples, this section focuses on a critical discussion of the results obtained, with a particular emphasis on the limitations of the presented technique.

As already mentioned, the effectiveness of the method depends, to a large extent, on the quality of sample excision. Each attempt to cut the sample with the use of oscillating knives resulted in the formation of shreds of cellulose fibers at the edges of the sample. Their presence obviously interfered with the effectiveness of the algorithms, mainly in the part that is responsible for recognizing the thickness of individual layers of paper in corrugated board. The second negative effect, which significantly affected the accuracy of the created algorithms, was damage to the sample before or after cutting, by the accidental crushing of the corrugated layer in the stochastic locations of the cross-section.

**Figure 16:**
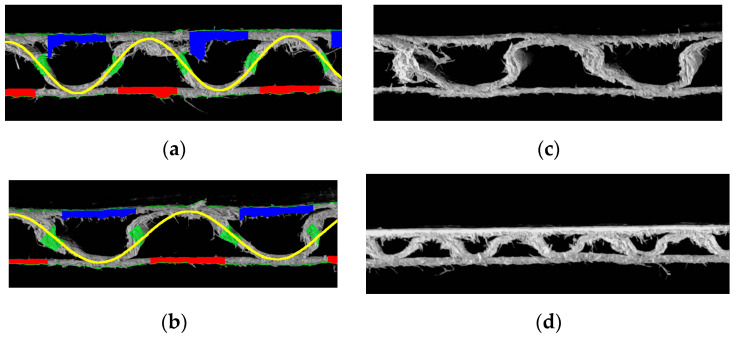
Examples of the corrugated board with damages: (**a**,**b**) irregular edges formed at the sample cut; (**c**,**d**) damages caused by crushing the corrugated layer.

In the case of the edge delamination of the paper or when an irregular edge was formed at the sample cut (example shown in Figure 16a,b), there was a clear difficulty in determining the correct thickness of the liners (flat layers), shown in Figure 16a, or the thickness of the flute (see Figure 16b). Damage to the corrugated board by crushing the corrugated layer obviously had the biggest impact on identifying the fluting period and amplitude (see examples shown in Figure 16c,d). Figure 16b also shows an inaccurate fit of the approximating function, which was assumed as a sine function, to the shape of the crushed fluting. However, despite the inaccurate adjustment of the approximating function to the real shape of the distorted corrugated layer, the period of the wave in most of the analyzed cases was determined with a good approximation. The amplitude in such cases was treated only as an indicator, determining the height of the cross-section after creasing.

**Figure 17:**
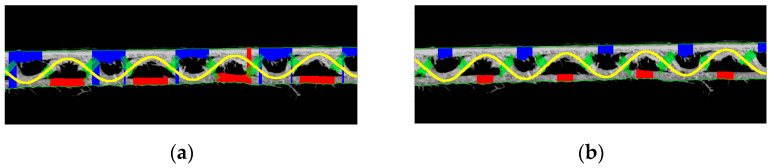
(**a**) The original example (weak effect) and (**b**) the operation on the same image after changing this area.

Unfortunately, almost all crushed microwave samples were only suitable for determining the wave period and amplitude (which are usually very well matched), while the problem was to determine the thickness of individual layers. This was due to too much interference (jagged fibers) in relation to the size of the area between the fluting walls. The solution that often allowed us to improve the efficiency of the algorithm was to limit the area on which the thickness of the liners was measured. The original example (weak effect) and the operation on the same image after changing this area are shown in Figure 17a,b, respectively.

In most cases, the source of the errors and failures of the algorithm for identifying the geometric parameters of the cardboard cross-section are paper fibers. Due to the fact that all samples were cut on a plotter with an oscillating knife, the disadvantage of which is precisely leaving this type of disturbance on the cutting edge, the task of the created program was significantly difficult. It can be suspected that cutting samples on a laser plotter would significantly improve the reliability of the results and would ensure an increase in the efficiency of the developed algorithm. However, the known edge-burning effect of the laser could also introduce other types of interference into the identification process, for example, leaving black soot on the cut edge, which could visually reduce the thickness of the papers in the analyzed cardboard samples.

In order to overcome all the above imperfections of the algorithm, we plan to apply artificial neural networks in future works on the identification of corrugated board types. Furthermore, the proposed algorithm can be utilized for other geometries, e.g., corrugated plastic sheets.

## 5. Conclusions

This article presents the procedure for identifying the geometric parameters of the cross-section of single-wall corrugated board. This procedure is based on a laboratory device developed for the purposes of this research, which was used to take pictures of cardboard samples in constant and controlled conditions. The developed procedure uses a genetic algorithm, which is characterized by a high efficiency and allows for very quick identification of the geometrical parameters of the corrugated board cross-section. The use of local algorithms based on, for example, gradients is unfortunately not possible in this case due to the specific formulation of the optimization problem.

Unfortunately, the effectiveness of the procedure is strongly dependent on the quality of the excised sample. In most cases, it was possible to correctly determine the wave period of the corrugated layer approximated by the sine function. The determination of the amplitude and thus also the adjustment of the approximating function to the fluting shape depended, to a large extent, on the level of cardboard cross-section crushing. Fluting parameters in samples without damage were identified in almost 100 percent of cases correctly.

However, the presented procedure is only the first step for automatic corrugated board type identification and modelling. It allows us to quickly find the main geometric characteristics of the corrugated board. In future, it can result in the possibility of performing numerical analyses, taking into account the real model of the corrugated board. Calculations made on a real (correctly mapped geometrically) model allow, for example, a more reliable analysis of the composition of the cardboard, and thus also the obtainment of greater savings in the optimization process.

## Figures and Tables

**Table 1:** Identification results of the geometrical features for the corrugated boards with flutes C, B and E.

	Flute C		Flute B		Flute E	
	[px]	[mm]	[px]	[mm]	[px]	[mm]
Flute height	187	3.60	140	2.70	67	1.30
Flute period	428	8.13	339	6.39	185	3.50
Board thickness	226	4.29	165	3.13	94	1.79
Upper liner thickness	27	0.51	24	0.46	21	0.40
Flute thickness	26	0.49	19	0.34	19	0.36
Bottom liner thickness	26	0.49	18	0.36	22	0.42

**Table 2:** Identification results of the geometrical features for the corrugated board with flute C (reference and crushed samples).

	Flute C (Reference)		Flute C (Crushed)	
	[px]	[mm]	[px]	[mm]
Flute height	187	3.60	157	3.00
Flute period	428	8.13	414	7.82
Board thickness	226	4.29	182	3.46
Upper liner thickness	27	0.51	27	0.51
Flute thickness	26	0.49	30	0.57
Bottom liner thickness	26	0.49	18	0.34

**Table 3:** Identification results of the geometrical features for the corrugated board with flute B (reference and crushed samples).

	Flute B (Reference)		Flute B (Crushed)	
	[px]	[mm]	[px]	[mm]
Flute height	140	2.70	118	2.20
Flute period	339	6.39	349	6.60
Board thickness	165	3.13	135	2.56
Upper liner thickness	24	0.46	23	0.44
Flute thickness	19	0.34	22	0.42
Bottom liner thickness	18	0.36	18	0.34

**Table 4:** Identification results of the geometrical features for the corrugated board with flute E (reference and crushed samples).

	Flute E (Reference)		Flute E (Crushed)	
	[px]	[mm]	[px]	[mm]
Flute height	67	1.30	56	1.10
Flute period	185	3.50	191	3.61
Board thickness	94	1.79	82	1.56
Upper liner thickness	21	0.40	14	0.27
Flute thickness	19	0.36	22	0.30
Bottom liner thickness	22	0.42	16	0.42

## Data Availability

Data are available on request.
